# Climate change exacerbates nutrient disparities from seafood

**DOI:** 10.1038/s41558-023-01822-1

**Published:** 2023-10-30

**Authors:** William W. L. Cheung, Eva Maire, Muhammed A. Oyinlola, James P. W. Robinson, Nicholas A. J. Graham, Vicky W. Y. Lam, M. Aaron MacNeil, Christina C. Hicks

**Affiliations:** 1 Changing Ocean Research Unit, Institute for the Oceans and Fisheries, The University of British Columbia, Vancouver, British Columbia Canada; 2 Lancaster Environment Centre, Lancaster University, Lancaster, UK; 3 Ocean Frontier Institute, Department of Biology, Dalhousie University, Halifax, Nova Scotia Canada; 4 Department of Mathematics and Statistics, Dalhousie University, Halifax, Nova Scotia Canada

**Keywords:** Environmental impact, Ecosystem services, Climate-change ecology

## Abstract

Seafood is an important source of bioavailable micronutrients supporting human health, yet it is unclear how micronutrient production has changed in the past or how climate change will influence its availability. Here combining reconstructed fisheries databases and predictive models, we assess nutrient availability from fisheries and mariculture in the past and project their futures under climate change. Since the 1990s, availabilities of iron, calcium and omega-3 from seafood for direct human consumption have increased but stagnated for protein. Under climate change, nutrient availability is projected to decrease disproportionately in tropical low-income countries that are already highly dependent on seafood-derived nutrients. At 4 ^o^C of warming, nutrient availability is projected to decline by ~30% by 2100 in low income countries, while at 1.5–2.0 ^o^C warming, decreases are projected to be ~10%. We demonstrate the importance of effective mitigation to support nutritional security of vulnerable nations and global health equity.

## Abstract (web summary)

The authors use fisheries databases and predictive models to understand past and future changes in the availability of iron, calcium omega-3 and protein from seafood. They show disproportional loss of nutrients in tropical low-income countries, which will be exacerbated by higher levels of global warming.

## Main

**Fig. 1:**
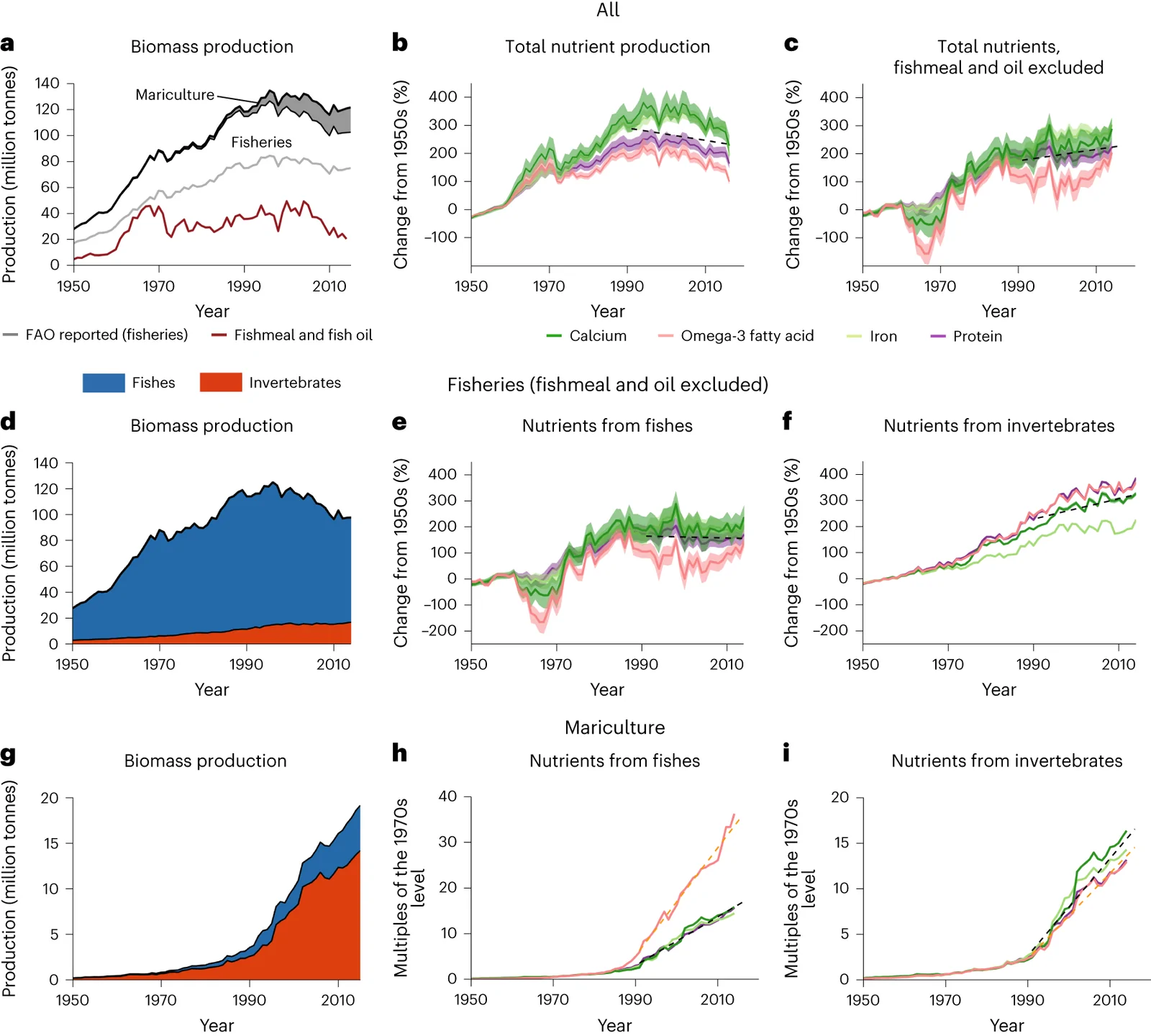
Historical changes in the supply of four nutrients (calcium, iron, omega-3 fatty acids and protein) from marine fisheries and mariculture production. **a**, Finfish and invertebrate production from marine fisheries catches and mariculture production. **b**,**c**, Changes in the four nutrients from global total fisheries catches relative to the 1950–1959 average (**b**) and from catches that excluded those for fishmeal and oil (**c**). **d**–**f**, Finfish and invertebrate production in weight from capture fisheries without those used for fishmeal and oil (**d**) and their estimated nutrient availability from fishes (**e**) and invertebrates (**f**). **g**–**i**, Finfish and invertebrate production in weight from mariculture (**g**) and their estimated nutrient availability from fishes (**h**) and invertebrates (**i**). Data from the United Nations’ Food and Agriculture Organization (FAO) fisheries statistics are included for comparison to Sea Around Us catch reconstruction data. Dotted lines represent average rates of changes in nutrient availability across the four nutrients estimated with linear regression models. Shaded bands represent the 95th percentile ranges from the uncertainties of the nutrient content estimation (including the assumption of edible portions and nutrient density of seafood) obtained from the Monte Carlo simulations (Methods). For mariculture production (**h**,**i**), separate lines were fitted to analyse the trends for the availability of omega-3 fatty acid (Methods).

In many low-income countries, marine fishes and invertebrates form an irreplaceable and affordable source of dietary micronutrients (for example, iron and zinc), vital to physical and mental development^1,2^. Consumption of fish is also promoted globally for health benefits such as protection against the risks of coronary heart disease and type II diabetes^3^. However, marine fisheries production peaked in the 1990s, with about 34% of the world’s fish stocks in 2017 classified as over-exploited and most of the remaining stocks as fully exploited^4^ (Fig. 1). During the same period, production from aquaculture of marine species (mariculture) has expanded rapidly to meet continued growth in demand for seafood^4,5^. Climate change has further affected exploited marine species through changes in species distributions and productivity, in turn altering catch composition^6–8^. These climate impacts are projected to continue through the twenty-first century, closely tracking emitted greenhouse gas levels^9,10^. Mariculture will also be affected by climate change through changes in environmental conditions, risks of diseases, harmful algal blooms and feed supplies from wild fish stocks^11–14^, raising concerns over the potential for mariculture to meet seafood demand.

Nutrient content varies considerably among marine species such that nutrient yields from fisheries catches are determined by catch composition and stock productivity^15,16^. However, it is unclear how changes in historic fisheries catch and mariculture production have altered the availability of important nutrients for human consumption and how future seafood nutrient availability will be impacted by climate-driven shifts in fisheries and mariculture production. Such information is critical for mitigating and adapting for climate risks to food security and, more broadly, developing priorities for investment to sustainably secure human health benefits from the ocean^17^.

Here we quantify past and future nutrient availability from seafood produced by global fisheries and mariculture (Methods). We focus on four nutrients that are plentiful in seafood and important to human health as an indicator of broader nutrient trends: calcium, iron, omega-3 fatty acids and protein. In many parts of the world, diets are lacking in calcium and iron, resulting in long-term health impacts^17^. Seafood is a key source of beneficial long-chain omega-3 fatty acids that are not readily available in other foods^18^, and protein has generally been the focus of work in fisheries for food provisioning^2^. This study combines global databases of fisheries catches and mariculture production^5,19,20^ with taxa-specific estimates of nutrient content in marine fishes and invertebrates to examine historical trends in seafood nutrient production (Methods). Using these datasets, we integrate projections from the latest generations of climate, fisheries and mariculture production models to examine the impacts of climate change and associated mitigation scenarios on global and regional seafood nutrient availability (Methods).

## Historical changes in nutrient availability from seafood

Globally, the total availability of calcium, iron, omega-3 fatty acids and protein from marine capture fisheries and mariculture increased from 1950, reaching a peak in the early 1990s, before declining substantially (Fig. 1a,b, *p* < 0.01; Supplementary Table 1). These declines from 1990 onwards, averaging −2.2% per year (0.8% per year, standard error) across all four nutrients (Fig. 1b), are largely a result of decreases in production from global finfish fisheries^21,22^. Meanwhile, a substantial amount of fisheries production was used as fishmeal and fish oil for livestock and aquaculture, and thus a smaller proportion of the nutrients produced from fisheries catches were available for human consumption (Fig. 1a). Global production from reduction fisheries for fishmeal and oil uses since the 2000s decreased (on average, 33% of total fisheries catches during this period; Methods and Fig. 1a). When we focused on nutrients that were available for direct human consumption by excluding the estimated catches from reduction fisheries for fishmeal and oil uses), the availability of iron, calcium and omega-3 fatty acids from seafood for direct human consumption increased (*p* < 0.01), although the availability of protein stagnated (*p* > 0.05) between the 1990s and 2010s (Fig. 1c,e; Supplementary Table 1 for the test statistics).

Nutrients made available from invertebrate fisheries and mariculture (including finfish and invertebrates) showed the largest increases from the 1990s (Fig. 1f,h,i and Supplementary Table 1). Yet these increases were not sufficient to substantially alter the overall trend of seafood-sourced nutrients because of the smaller contributions of invertebrate fisheries and mariculture to total seafood production (Fig. 1d,g). This is further compounded by the relatively low edible portion of the total wet weight of shellfish (28–56% for molluscs (except cephalopods and krill) and crustaceans, respectively) relative to finfish (33–92%)^23^ (Methods). In addition, the increasing production of finfish and crustaceans from mariculture that are largely fed using aquafeed also increased the demand for fishmeal and fish oil. For example, in 2007 and 2008, aquaculture consumed 3,844,000 tonnes of fishmeal (68.4% of total aquafeed production) and 782,000 tonnes of fish oil (73.8% of total production)^24^. Although 70% of mariculture comes from farming bivalves that do not require aquafeed inputs, the total volume of finfish and crustaceans that require aquafeed increased about 50 times between 1990 and 2010, driven by growing demand in high-income countries for high-value omnivorous and carnivorous species^4,5^.

## Potential seafood nutrient availability under climate change

**Fig. 2:**
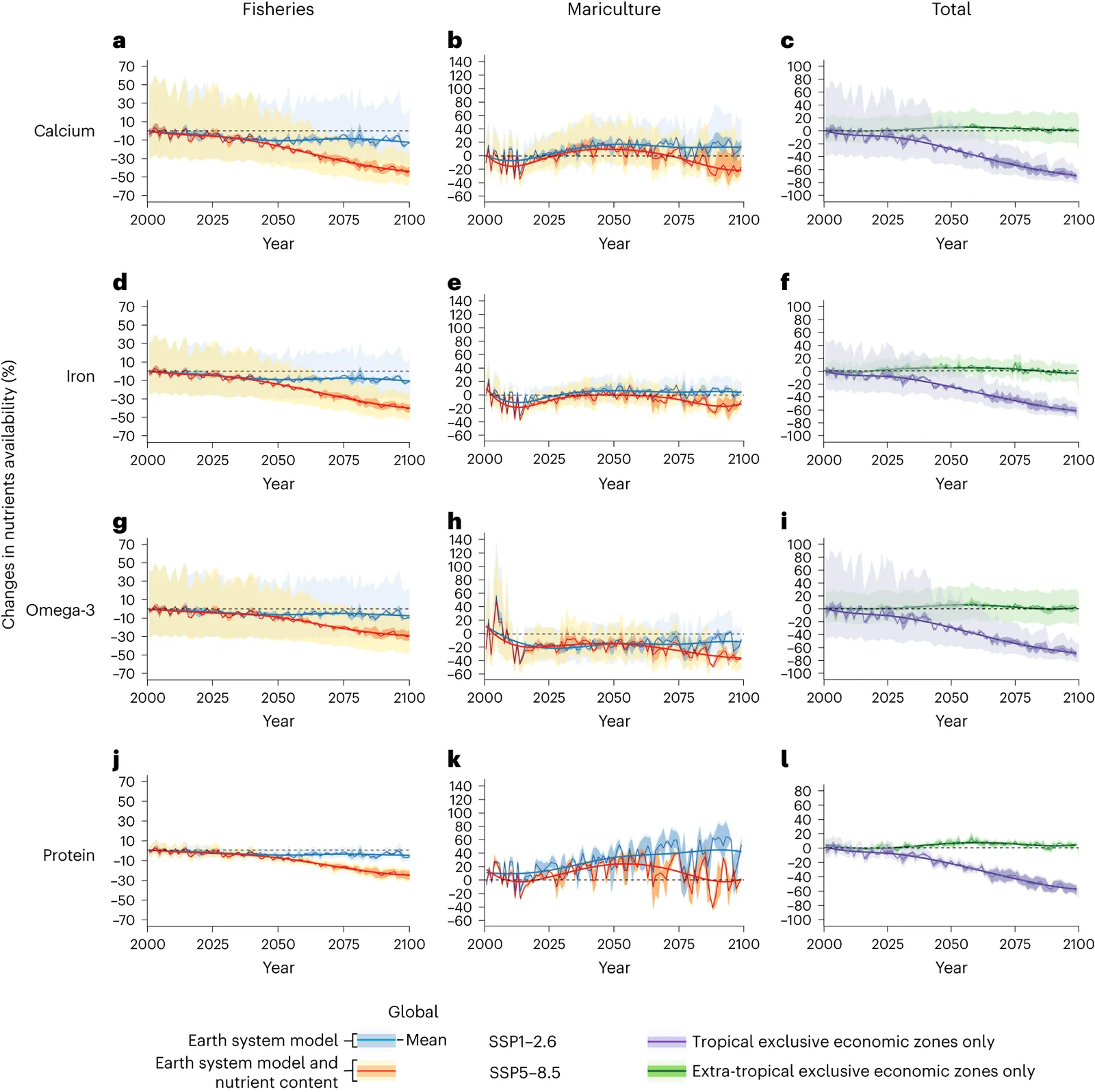
Projected changes in the availability of calcium, iron, omega-3 fatty acids and protein from global potential fish and invertebrate production for the twenty-first century under climate change scenarios. **a**–**l**, The projections are subdivided by marine fisheries (**a**,**d**,**g**,**j**) and mariculture (**b**,**e**,**h**,**k**) under SSP1–2.6 and SSP5–8.5 and the combined production potential from fisheries and mariculture sub-divided by tropical and extra-tropical Exclusive Economic Zones under SSP5–8.5 (**c**,**f**,**i**,**l**). Solid lines represent estimates using the mean nutrient contents of fisheries and mariculture production potential across Earth system models. Blue and orange lines and shading represent SSP1–2.6 and SSP5–8.5 scenarios, respectively. The lighter shaded bands represent the maximum and minimum range of projections calculated from the Earth system models and the 95th percentile ranges from the uncertainties of the nutrient content estimation (including the assumption of edible portions and nutrient density of seafood) obtained from the Monte Carlo simulations (Methods). The darker shaded bands represent the uncertainties associated with the Earth system models only.

Globally our integrated climate–fisheries–mariculture model projected decreases in the availability of calcium, iron, omega-3 fatty acids and protein from potential marine fish and invertebrate catches in the twenty-first century relative to the present day (Fig. 2; Methods). By 2050, fisheries production of all four nutrients is projected to decrease by 5 to 10%, relative to levels in 2000 under ‘strong mitigation’ low greenhouse gas emissions (Shared Socio-economic Pathway (SSP)1–2.6) and 8 to 15% under ‘no mitigation’ high emissions (SSP5–8.5) scenarios (Methods) (Fig. 2a,d,g,j and Supplementary Tables 2 and 3). The gap in nutrient availability between these scenarios widened substantially by 2100, with minerals (calcium and iron) showing the largest projected declines under SSP5–8.5. The availability of calcium and iron from fisheries is projected to decline by 41% (36–45%) and 37% (32–41%) by 2100, respectively, under SSP5–8.5 (Fig. 2a,d). Such declines are lower under the ‘strong mitigation’ scenario (around 10% for both calcium and iron by 2050 and 2100). Given there is already an inadequate dietary supply of calcium for almost half of the world’s population^21^, the projected decrease in calcium from seafood will probably intensify the risk of deficiency in the future. The decrease in availability of omega-3 fatty acids (decrease by 22–31% by 2100; Fig. 2g) and protein (decrease by 18–25% by 2100; Fig. 2j) under SSP5–8.5 are comparatively less sensitive than calcium and iron throughout the twenty-first century.

The relatively larger declines in mineral micronutrients (calcium and iron) from fisheries under SSP5–8.5 than protein and omega-3 fatty acids is largely driven by the projected decreases in maximum catch potential of pelagic fishes, particularly in the tropics (Supplementary Tables 2 and 3). These pelagic fishes, especially the small- to medium-sized species that include anchoveta, sardines and mackerels, are highly productive and rich in calcium and iron^15^. While pelagic fishes accounted for almost one-third of total catches in the 2000s, their maximum catch potential is projected to decrease by 46 to 60% (inter-model range) by 2100 under SSP5–8.5. Although invertebrates also have high mineral content and decrease in their catch potential is projected to be relatively small (~2% by 2100 relative to 2000 under SSP5–8.5), they represent a substantially smaller proportion of total catches and have a smaller proportion of edible biomass that would not allow them to compensate for global nutrient declines.

More calcium, iron and protein are projected to be available from mariculture production by 2050 relative to 2000, while the potential availability of omega-3 fatty acids is projected to decrease (Fig. 2b,e,h,k; Methods). In particular, 11 to 23% more calcium and 18 to 45% more protein are projected to be available from mariculture by 2050 relative to 2000 across both emissions scenarios. Invertebrate mariculture production contributed most to the supply of calcium and protein in the recent past, and these supplies are projected to remain at a similar level by 2050 (Supplementary Tables 4 and 5). Simultaneously, total potential mariculture production from non-invertebrate groups is projected to increase by 7–19% by 2050 across both emissions scenarios. Yet calcium and protein from mariculture production are likely to be volatile as it is largely contributed by invertebrate production that is strongly affected by fluctuations in farm-gate prices (Fig. 2b,k; Methods). Our model projected a decline in the availability of omega-3 fatty acids relative to 2000 due to decreased large benthopelagic and demersal finfish mariculture production potential that have high omega-3 fatty acids content (Fig. 2h). However, availability of all four nutrients remains relatively stable between 2050 and 2100 under SSP1–2.6 but decreases rapidly under SSP5–8.5 during this period. Our analysis focuses on potential changes in countries with established mariculture production only; scenarios of new mariculture development in other countries would also be expected to increase nutrient production from farmed species. Also, we did not consider scenarios of changes in mariculture systems and technology, for example, aquafeed formula.

Mariculture production presently contributes around 15% of total seafood production and 10–25% of the availability of the associated four nutrients (Supplementary Fig. 1), yet our projected increases in mariculture production across the four nutrients are not able to compensate for the projected loss from fisheries globally in the twenty-first century. Under the ‘strong mitigation’ scenario, increases in nutrient availability of calcium, iron and protein from mariculture by 2050 are dwarfed by decreases in their availability from capture fisheries. Under the ‘no mitigation’ scenario, by the end of the twenty-first century, any gains in nutrient availability from potential mariculture production before the mid-twenty-first century are lost. For calcium, iron and omega-3 fatty acids, projected declines in mariculture production potential would result in a net-loss of 20–30% by 2100 relative to the 2000s.

## Regional disparities in nutrient availability

Whereas global nutrient declines are of substantial concern, these projections hide large regional disparities in the future impacts of climate change on the availability of seafood-sourced nutrients (Fig. 2c,i,f,l). For Exclusive Economic Zones (EEZs) centred in the tropics (between 23.5° N and S latitude), availabilities of all four nutrients from fisheries and mariculture are projected to decline by 22–25% and 51–61% by 2100 relative to 2010, under the SSP5–8.5 scenario. In contrast, nutrient availability from extra-tropical (that is, outside the tropics) EEZs is projected to experience small gains throughout the twenty-first century. Moreover, nutrients from mariculture production are projected to become more important in the tropics under SSP5–8.5 because of their higher climate resilience relative to capture fisheries (Supplementary Fig. 2). In extra-tropical regions, however, the projected relative contribution of mariculture to the availability of the four nutrients does not change substantially in the twenty-first century under the two climate scenarios. These disparities, along with a dramatic decline in marine nutrients in the tropics, are of particular concern given that dietary nutrient deficiencies are greatest in tropical regions^25^.

**Fig. 3:**
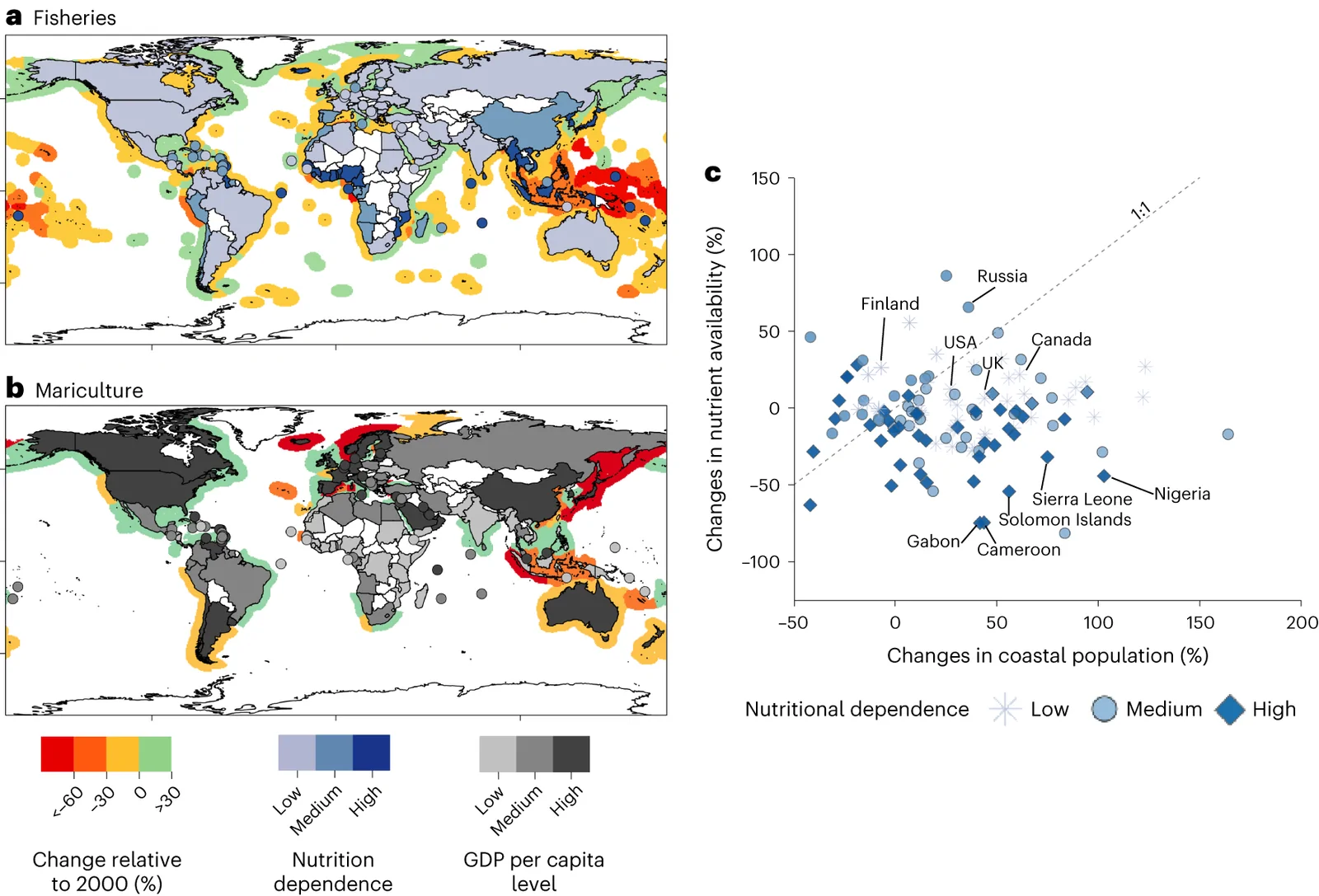
Projected changes in available seafood-sourced nutrients by countries across calcium, iron, omega-3 fatty acids and protein by 2050 relative to 2010 under the SSP5–8.5 scenario. **a**,**b**, Average projected changes in nutrients by EEZs of maritime countries from fisheries and mariculture, respectively. EEZs without mariculture production in the present day are not coloured. The colours of the land area represent an index of nutritional dependence on seafood (**a**) and gross domestic products (GDP) per capita (**b**) in 2010 by country. On the maps, for countries with small land area, nutritional dependence (**a**) and GDP per capita (**b**) levels are shown in dots to increase their visibility. The colours in the ocean area represent projected changes in nutrients by EEZs. **c**, Projected changes in nutrient availability by country (average across the four nutrients) by 2050 relative to 2010 in relation to the projected changes in coastal population during the same period under SSP5. The dotted line indicates the projected changes in nutrient availability equal to the changes in coastal population. Countries above the dotted line have projected changes in nutrient availability that are less than changes in coastal population and vice versa. Icons and colour scale indicate the estimated nutritional dependence of the countries (Methods).

The tropical versus extra-tropical disparity in climate impacts on nutrient availability is particularly strong for marine fisheries. EEZs in the tropical Pacific region are ‘hotspots’ of decline, with projected loses of >30% (Fig. 3a) and some projected to lose more than 60% of their available nutrients from fisheries catches by 2050 under the ‘no mitigation’ scenario. Such declines are driven by large projected decreases in biomass and in some extreme cases local extinction of exploited species due to poleward range shifts^9^. Many areas with large projected declines in nutrient availability fall within the EEZs of countries that are currently strongly dependent on seafood as a source of nutrients, particularly in Southeast Asia, Pacific Island nations and West Africa^26–28^ (Fig. 3). Moreover, specific hotspots, such as the EEZs of Indonesia, are projected to experience additional nutrient losses from declines in mariculture production (Fig. 3b). However, the greatest mariculture losses are expected in high production areas, such as Australia, Chile, China, New Zealand and Norway, where people are less dependent on seafood (Fig. 3a). Projected increases in mariculture potential in some of these hotspots, such as those in the South China Sea, are not able to compensate for the loss in fisheries potential.

Most countries with projected declines in nutrient availability are also facing increases in population size that could exacerbate nutritional risks to people (Fig. 3c). Particularly, under the SSP5-8.5 scenario, nutrient availability per capita is projected to decrease in the majority of countries. This contrast between declines in nutrient availability and population growth is especially large in countries such as Nigeria, Sierra Leone and the Solomon Islands, where coastal populations are projected to increase by over 50% by 2050 relative to 2010 (Fig. 3c). In contrast, high latitude countries such as Finland and Russia are projected to increase in nutrient availability from fisheries and mariculture beyond their population changes.

## Relating seafood nutrient availability to global warming levels

**Fig. 4:**
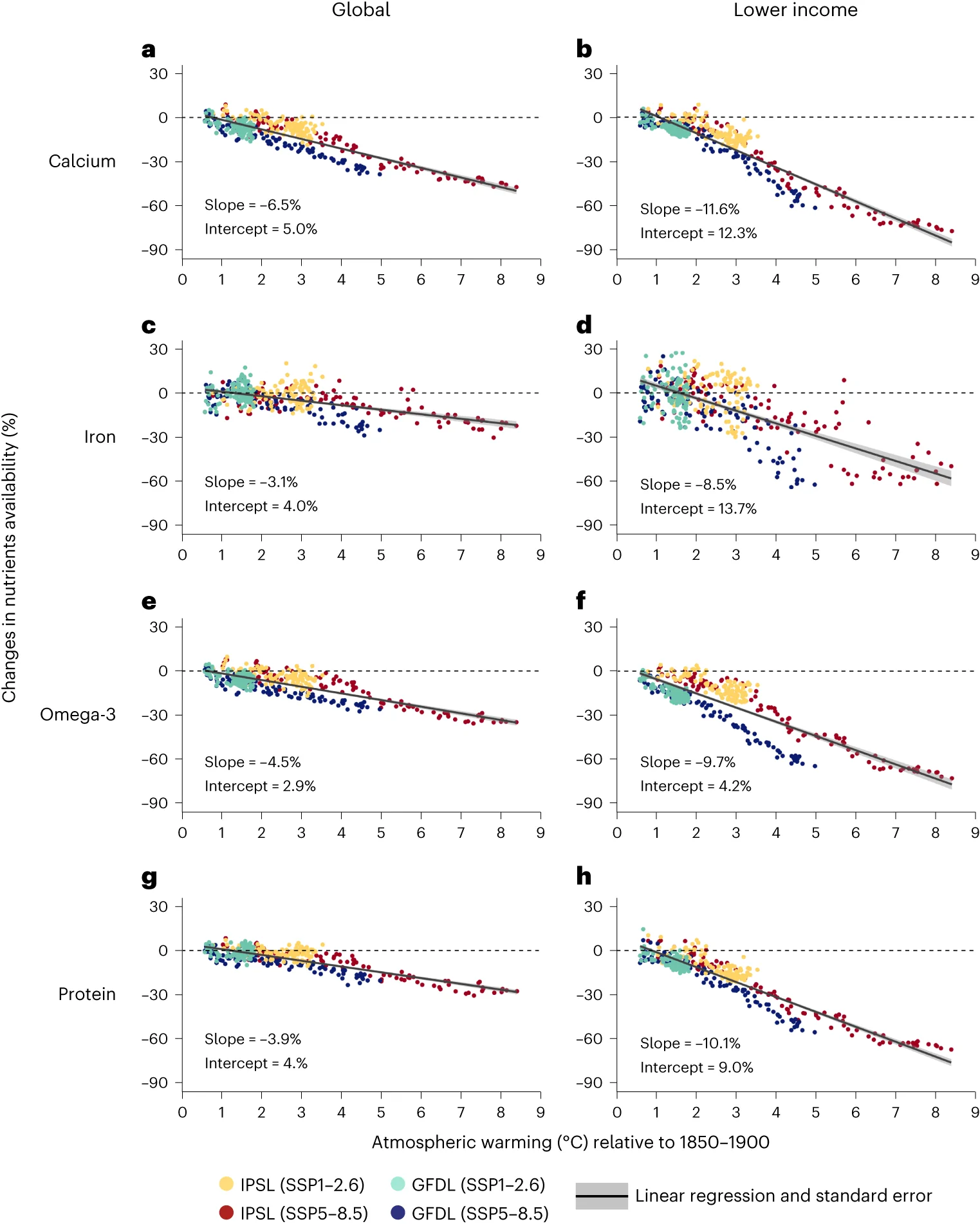
Scaling between projected atmospheric warming and changes in the availability of calcium, iron, omega-3 fatty acids and protein from fisheries catches and mariculture production globally and in lower-income countries. Atmospheric warming is represented by global surface air temperature relative to pre-industrial (1850–1900) levels. Each data point represents an annual projected nutrient availability driven by outputs from a specific Earth system model and climate change scenario relative to 2000. The lines are based on linear regressions, with the estimated slopes and intercepts noted in each panel. Lower-income countries refer to those with gross domestic product per capita (2010) below the median value of all maritime countries (Methods). **a**,**c**,**e**,**g**, Global changes in calcium (**a**), iron (**c**), omega-3 (**e**) and protein (**g**). **b**,**d**,**f**,**h**, Lower-income country changes in calcium (**b**), iron (**d**), omega-3 (**f**) and protein (**h**).

Our projected declines in nutrient availability from fisheries and mariculture scale significantly and linearly with levels of atmospheric surface warming (Fig. 4, *p* < 0.05 and Methods). Globally, seafood-sourced nutrient availability is projected to decrease at a rate of 3.1–6.5% per degree Celsius of warming relative to pre-industrial levels (Fig. 4a,c,e,g). Among the four studied nutrients, the availability of calcium from fisheries and mariculture production is most sensitive to global warming (6.5 ± 0.2% standard error (s.e.) per degree Celsius of warming). However, for lower-income countries (below median GDP per capita globally; Fig. 3b) the rate of decline in nutrient availability per unit of warming is two to three times the global average (9.7–11.6% per degree Celsius of warming, Fig. 4).

## Discussion

Our results highlight that global nutrient availability for direct human consumption from capture fisheries have been stagnant in the past few decades. The increasing utilization of fisheries production by reducing the reliance on fishmeal and oil for aquaculture and livestock production, such as through the increasing uses of non-seafood-sourced feed formula^29,30^, could increase nutrient availability from seafood for human consumption that could compensate, to some extent, the impacts of climate change on fisheries and aquaculture production. Regionally, low-income countries, which often depend more on fish as an important source of nutrients, will face much bigger challenges to food security if the world fails to achieve the international agreement to keep global warming well below 2 °C of pre-industrial levels. For example, without effective policy to mitigate greenhouse gas emissions, global atmospheric warming is expected to reach a level beyond 4 °C above pre-industrial levels by 2100. For lower-income countries, this level of climate change corresponds to a projected ~30% decline in calcium, iron, omega-3 and protein availability from fisheries and mariculture by 2100 (Fig. 4). Even if the climate is stabilized closer to the 1.5–2.0 °C global warming targets specified in the Paris Agreement, risks remain (~10% decrease in nutrient availability) for lower-income countries. These countries would also need to adapt to the projected impacts from any transient ‘overshoot’ of warming before global climate mitigation targets can be met. In addition, higher-income countries projected to experience impacts to seafood production may adapt by increasing nutrient availability through importing seafood produced elsewhere. Such trade disparities may increase international competition for a limited seafood supply, further challenging lower-income countries to fill widening gaps in nutrient availability under climate change^31^. This demonstrates the importance of coordinating actions and global responsibility towards climate mitigation and food security. Given that many coastal developing countries rely on the ocean for achieving sustainable development goals, the added challenges from climate change will increase the uncertainties of achieving these important societal targets^32^.

Projected disparities in nutrient availability could be reduced by developing nutrient-sensitive management approaches that account for climate-driven impacts to capture fisheries and mariculture. Particularly for countries where seafood nutrient availability is at risk under climate change, marine fisheries and mariculture should incorporate goals and strategies that can help fill the increasing gaps in nutrient production. Adapting fisheries management to account for species range shifts and productivity is expected to increase fishing yields and profits^33^, and, similarly, implementation of nutrient-based fisheries governance^34^ and management^35^ approaches would limit projected impacts to nutrient availability. Managing and developing fisheries and aquaculture for nutrients also provides a basis for integrating nutrient information into climate-resilient ocean planning, such as designing fishing zones that maintain long-term nutrient yields given projected shifts in species distributions^36^. Moreover, enabling countries with nutrient-deficient populations to prioritize sustainable fishing and aquaculture (including freshwater) of the most nutritious and less climate-sensitive species could help reduce current global disparities in nutrient availability^37^.

Seafood production from aquaculture has been projected to increase towards the mid-century^38^ depending on climate and socio-economic scenarios. This increase might contribute substantially to the available nutrition by 2050. Moreover, climate-smart adaptation strategies such as fishmeal replacement with alternative sustainable protein can lower climate impacts on a subset of finfish production and increase mariculture production by 25%–42% (ref. ^38^). Furthermore, future aquaculture growth that attends to the diversity of species produced, nutrient access of poor consumers and environmental sustainability can contribute to increasing nutrient security particularly for low- and middle- income countries^39^. Tropical freshwater fish, which are not included in our analysis, are an important source of nutrients in many parts of South America, Africa and Asia^40–42^. However, the potential of these nutrient sources relies on the sustainable and climate-resilient development of aquatic food systems^43,44^.

This study paints a picture of challenging and uncertain futures regarding equitable availability of seafood-sourced nutrients in the world under climate change. In addition to uncertainties around pathways of climate mitigation, seafood nutritional content varies within and between species and are affected by climate-mediated changes in primary productivity, fish metabolism and foodwebs that may vary between different ecosystems and regions^45–48^. For example, temperature can affect the uptake of micronutrients such as iron and calcium^49^ and the synthesis of omega-3 fatty acids^50^ by aquatic animals. Overall, limited availability of seafood nutritional content data and studies by species, regions and production systems are a challenge for more accurate estimation of seafood nutrient availability from fisheries and mariculture.

In addition, availability of nutrients from seafood production for human consumption is determined by many non-climatic factors, such as seafood processing, dietary choices, culture, food prices, trade and seafood sourcing. For example, analysis of global catch, trade and nutrient composition data suggests that foreign fishing and international trade divert nutrients caught in marine fisheries from nutrient-insecure towards nutrient-secure nations^51^. Edible portion further determines availability of nutrients from seafood but varies widely across species, regions and consumers’ habits. For example, across Africa and Asia, many species are eaten whole^52,53^, whereas across Europe, supply could be increased by more efficient processing^54^, or the adoption of practices that do not discard nutrient-dense components (for example, fish heads, bones)^55^. The limited number of estimates on edible portions of seafood exacerbates the uncertainties in understanding the contribution of seafood to human nutrition under climate change. Future studies considering scenarios of these other non-climatic human drivers on seafood nutrient availability would help elucidate the relative contributions of these scenario uncertainties to the future of seafood nutrient availability. Further work is needed to better understand the effects of changing environment on the nutritional content of exploited marine species and to develop and explore scenarios of social and economic changes on global and regional seafood nutrient security.

Though the large-scale patterns of projected changes in catch and mariculture production potential from our models agree with previous estimates, the magnitude of climate impacts vary between models, particularly in the Arctic Ocean^56^. Moreover, ocean conditions projected by earth system models do not fully represent coastal oceanographic processes that are important to some fisheries and mariculture production, such as those in eastern boundary upwelling systems. Notwithstanding the uncertainties of climate-fisheries projections, the key findings of this study, including at-risk regions, the global disparity in nutrient availability under climate change and the implications for nutrient-sensitivity fisheries and mariculture, are generally robust to the uncertainties of the nutrient content and assumption of the edible proportion of the exploited species (Fig. 2 and Supplementary Tables 2 and 5). Our results provide a foundation for higher-resolution regional analyses to build upon.

## Conclusions

There is a growing emphasis on the critical role seafood can and must play in tackling food security and transitioning to sustainable diets^15,17^. Here we have demonstrated that nutrient availability from global fisheries has been declining since 1990 and is projected to decline further under climate change. Increases in mariculture will only partly compensate for these losses. The greatest losses of nutrients from seafood lie in tropical and low-income countries where current and future nutritional needs are greatest, resulting in global disparities in nutrient availability under climate change. Moreover, climate change can impair not only nutrient availability from global fisheries but is also expected to impact agriculture production and reduce iron, zinc and protein concentrations of crops^57^. Thus, limiting warming to under 2 °C is critically important to reduce nutrient losses from agriculture and seafood sectors, especially in tropical and low-income countries that are most likely to be severely impacted. Our results highlight the need for nutrition- and climate-sensitive fisheries management, with food-based trade policies developed to prevent the changes we predict in nutrients from seafood translating to substantial malnutrition and declines in public health^51^.

## Methods

### Climate scenarios and Earth system models projection

We used outputs from two Earth system models that participated in the Coupled Model Intercomparison Project Phase 6 (CMIP6) under two contrasting emissions scenarios. The two Earth system models included Geophysical Fluid Dynamics Laboratory (GFDL)-ESM4 and the Institut Pierre-Simon Laplace (IPSL)-CM6A-LR. The variables that we extracted from the Earth system models include mean surface temperature, sea surface and bottom temperature, oxygen concentration and salinity, net primary production, sea ice extent and surface advection. Projections are under two contrasting scenarios—SSP1—Representative Concentration Pathway (RCP) 2.6 (SSP1–2.6) and SSP5–8.5. The SSP1–2.6 and SSP5–8.5 scenarios represent a ‘strong mitigation’ low-emissions pathway and a ‘no mitigation’ high-emissions pathway, respectively. Under the SSP1–2.6 and SSP5–8.5 scenarios, global warming levels are projected to be limited to 2 °C and exceeding 4 °C by 2100 relative to 1850–1900, respectively^58^.

### Edible portion

Using data available from published literature, we used estimates of edible portion for different classes of seafood and converted liveweight into estimates of edible seafood mass and calculated nutrient availability of these seafood for human consumption. Our estimates of edible portion of marine fishes and invertebrates were based on those from: (1) United Nations’ Food and Agriculture Organization (FAO)^59^, (2) FAO/INFOODS^60^ and (3) the Aquatic Foods Composition Database^61^ (Supplementary Table 6). Edible portion varies considerably across species and geography^50^. To account for this variation, we grouped the edible portion estimations for each set by functional groups that are defined by species’ body size and ecology (Supplementary Table 6) and calculated the average, minimum and maximum values from these datasets.

### Nutrient content of fishes and invertebrates

We estimated calcium, iron, omega-3 fatty acids and protein content in fishes and invertebrates using a database of published nutrient composition studies, including 419 finfish species and 63 invertebrate species that represent a substantial proportion of the production from fisheries and mariculture (40% and 60% of production by weight, respectively). For fish, nutrient content was estimated using a trait-based Bayesian model that was developed to quantify nutrient yields from global marine fisheries^15^ (Hicks et al.^15^; https://github.com/mamacneil/NutrientFishbase). Our model predicts nutrient concentrations according to species-level information using phylogenetic hierarchy (order > family > genus > species), diet (trophic level, feeding pathway), thermal regime (tropical, sub-tropical, temperate, polar) and energetic demand (body size, growth rate, age at maturity, body shape), enabling out-of-sample predictions for species that do not have published nutrient content data based on their traits and the lowest-level phylogenetic intercept available from those in the observed data. We predicted nutrient concentration for calcium (mg 100 g^−1^), iron (mg 100 g^−1^), omega-3 fatty acids (g 100 g^−1^) and protein (%) of muscle tissue for each marine fish species in the Sea Around Us catch dataset, using traits extracted from Fishbase^62^.

For invertebrates, 63 species in the nutrient composition database (195 samples in 50 studies) represented all fished and farmed invertebrate classes in the catch projection dataset (bivalvia, cephalopoda, gastropoda, malacostraca). We assigned nutrient values to landed invertebrate catch by estimating the mean calcium, iron, omega-3 fatty acids and protein concentration of edible meat for each invertebrate genera, family, order and class in descending order of priority when data were available. Calcium and iron values were available for bivalvia and malacostraca (73% of fished species) and omega-3 fatty acids values were available for bivalvia, malacostraca and cephalopoda (80% of fished species). Protein values were available for all invertebrate classes. Species without class-level nutrient data were assigned the mean nutrient concentration estimated from their closest taxonomic groups. Because of the limited data, we assume that farmed and wild species have the same nutrient content, although the nutrients content of farmed species would be dependent on their feed and farming systems.

### Historical fisheries and mariculture data

We obtained catch data from the *Sea Around Us* (SAU) reconstruction database (www.seaaroundus.org). We also extracted the marine capture production data (tonnes) of each country and species from 1991 to 2018 from the Food and Agriculture Organisation of the United Nations (FAO) using the latest version of FishStatJ of the FAO’s Fisheries and Aquaculture statistics. Because the reconstructing catch data provided by the SAU database utilized a wide variety of data sources and information to estimate all of the fisheries components such as subsistence catch, recreational catch and discards that are missing from the official reported data^19^, we used this set of data in our analysis to capture a more comprehensive estimation of the total availability of nutrients from marine fisheries. Annual catch data were extracted from the *Sea Around Us* database of reconstructed catches, which cover the years 1991 to 2016, distributed onto 180,000, 30′ latitude × 30′ longitude spatial cells of the world ocean^63^. The catch allocation process by the SAU produced spatial time series of landings data from 1991 to 2016 that were aggregated into different fishing entities and which distinguished between landings by different taxa, different fishing gear types, between distant-water and domestic fleets, different catch types (landings and discards) and between different fishing sectors (including industrial, subsistence, artisanal and recreational). We also used published estimates of catches by species that were used in reduction fisheries to produce fishmeal and oil for each year^64^. We assumed that nutrients from these reduction fisheries were not available for direct human consumption.

We used an updated version of the Sea Around Us Global Mariculture Database (GMD) (www.searoundus.org)^5,20^. Mariculture (marine aquaculture, including brackish aquaculture) production in the database includes marine and brackish aquaculture production by taxa by each province or state for each maritime country from 1950 to 2015. GMD contains over 307 farmed species in both marine and brackish water produced in 112 maritime countries and territories with a total of 656 provinces or states. The database includes published data from national and provincial/state-level reports and the FAO.

### Human population within a 100 km coastal band

To calculate coastal population, we created a 100 km buffer along the coastline of each country based on the Global Administrative Areas database (GADM v.2.8) and used this to intersect with the Global 1-km Downscaled Population Grids; https://sedac.ciesin.columbia.edu/data/set/popdynamics-1-km-downscaled-pop-base-year-projection-ssp-2000-2100-rev01/data-download) to calculate projected changes in coastal populations for 2050 relative to 2010 under SSP5.^65^

### Projecting fisheries catch potential

We used the Dynamic Bioclimate Envelope Model (DBEM) to project changes in potential catches of exploited marine fishes and invertebrates^66^. The current distributions of relative abundance of commercially exploited species (representing 1970–2000) were predicted on a 0.5° latitude × 0.5° longitude grid. The distribution was predicted based on the species’ depth range, latitudinal range, polygons encompassing their known occurrence regions. The distributions were further refined by assigning habitat preferences to each species. The required habitat information was obtained from FishBase (www.fishbase.org) and SeaLifeBase (www.sealifebase.org). Index of habitat suitability was computed for each species in each spatial cell from temperature (bottom and surface temperature for demersal and pelagic species, respectively), bathymetry, specific habitats (coral reef, continental shelf, slope and seamounts), salinity (bottom and surface temperature for demersal and pelagic species, respectively) and sea ice with 30-year averages of outputs from 1971 to 2000 from the two Earth system models. Movement and dispersal of adults and larvae were modelled through advection–diffusion–reaction equations. Carrying capacity in each cell is assumed to be a function of the unfished biomass of the population, the estimated habitat suitability and net primary production in each cell. The maximum sustainable yield of the species was approximated by the average of the top ten annual catches^67^. The average mass of an individual in the cell was simulated using a sub-model derived from a generalized von Bertalanffy growth function. The DBEM had a spin-up period of 100 years using the climatological average oceanographic conditions from 1971 to 2000, thereby allowing the population to reach equilibrium before it was perturbed with oceanographic changes. Biomass and catch were then calculated from the population mean body weight and abundance. To calculate maximum catch potential, the fishing mortality rate is set to be equal to the natural mortality rate.

### Projecting mariculture production potential

To project future mariculture and quantify the impact of climate change on its production, we used the global mariculture production model (GOMAP)^20,65,68^. The model projects changes in mariculture production potential (MPP) by accounting for changing ocean conditions, suitable marine area for farming, fishmeal and fish oil production, the dietary demand of farm species, farmed gate price, global seafood demand and characteristics of the farm species to under two combined emissions and socioeconomic scenarios. MPP is defined as the maximum amount of biomass of a species that could potentially be continuously farmed for decades at a particular marine area.

The GOMAP is a four-step framework to project MPP under different climate change and socioeconomic scenarios. First, for each farmed species, the model predicts the marine areas within the EEZ where it would be suitable for mariculture activities and uses species distribution models to quantify the ecological niche of each species for the present-day period (1991–2010) and calculated habitat suitability index (HSI) for each 0.5° latitude by 0.5° longitude grid cell of the ocean. The model then applied spatial filters that were informed by physical and social-economic constraints of mariculture location to generate a potentially suitable area for mariculture and further applied species distribution models to project future HSI and qualify the potential suitable marine area for mariculture under climate change. Second, with the integrated Artificial Neural Network algorithm for price forecasting, GOMAP projects farm gate prices for each farm species per each EEZ. Third, with the Feed Formulation Model, the model estimates the amount of Fishmeal required for species production and quantified the total crude protein index. For this study, we used forage fish species’ total catch in weight used in reduction fisheries for each year^64^. For the projected period (2015 to 2100), the model assumed a constant forage fish usage as the recent five-year average percentage (2010–2014). Lastly, using a general additive model (GAM), an empirical relationship was developed with current suitable marine areas for mariculture, the species’ farm gate price, HSI for mariculture and total crude protein index to estimate the mariculture production potential.

### Calculating changes in seafood nutrient availability

We calculated changes in the availability of the four nutrients, calcium, iron, omega-3 fatty acids and protein, from fisheries and mariculture in the past by using the estimated nutrient content of exploited species and their reported catches and mariculture production from 1950 to 2016. For future projections, we calculated the projected maximum catch potential and mariculture production potential from DBEM and GOMAP, respectively, for each studied exploited species globally and by exclusive economic zones. We next calculated the annual percent changes in maximum catch potential and mariculture production potential relative to the reference period (1991–2010). We then multiplied the relative changes in production potential to the reported production of the species during the same period and then calculate the changes in total fisheries and mariculture potential globally and by exclusive economic zones under the two scenarios (SSP1–2.6 and SSP5–8.5). We calculated the confidence intervals of the projected changes in nutrients availability using Monte Carlo simulations to sample values of nutrient content of seafood species (*N* = 1,000) based on the mean and 95 percent intervals of the estimates and the estimated edible portion by functional group based on the available minimum and maximum values. We used the median values of the estimated nutrient availabilities of the reference periods as baselines and expressed the results from each iteration of the Monte Carlo simulations relative to such baselines. Thus, the calculated confidence intervals also represented the variations in the absolute values of the estimated nutrient availability due to uncertainties of the nutrient content and edible portion of seafood.

## Online content

Any methods, additional references, Nature Portfolio reporting summaries, source data, extended data, supplementary information, acknowledgements, peer review information; details of author contributions and competing interests; and statements of data and code availability are available at https://doi.org/10.1038/s41558-023-01822-1.

## Supplementary information

## Data Availability

All the data presented in this manuscript are made available through Dryad at https://doi.org/10.5061/dryad.b5mkkwhdd.

## Code availability

The code required to reproduce the results presented in this paper are availability at https://doi.org/10.5061/dryad.b5mkkwhdd.
