# Supplementary material for: Climate change exacerbates nutrient disparities from seafood
Source: Nat Clim Chang. 2023 Oct 30;13(11):1242–9. doi: 10.1038/s41558-023-01822-1 (PMC10624626; doi:10.1038/s41558-023-01822-1)
Supplement: Supplementary file 1 — Supplementary Tables 1–6 and Figs. 1 and 2. [file 41558_2023_1822_MOESM1_ESM.pdf]

nature climate change

Article https://doi.org/10.1038/s41558-023-01822-1

Climate change exacerbates nutrient disparities from seafood

In the format provided by the authors and unedited

Supplementary Tables

Table S1. Test statistics of the linear regression between the average changes in production across the four nutrients (calcium, iron, omega-3 fatty acid and protein) from fisheries and mariculture between 1990 and 2016.

Dataset	Coefficient	Estimate	Standard error	t value	p-value
Nutrients from total fisheries and mariculture production	Intercept	4759.1%	1596.4%	2.98	0.0036
	Year	-2.2%/year	0.8%/year	-2.76	0.007
Nutrients from fisheries and mariculture production excluding fishmeal and oil	Intercept	-3715.4%	1631.7%	-2.3	0.025
	Year	2.0%/year	0.82%	2.5	0.016
Nutrients from fishes produced from capture fisheries (excluding fishmeal and oil)	Intercept	1036.2%	1548.3%	0.67	0.505
	Year	-0.39%/year	0.77%/year	-0.50	0.617
Nutrients from invertebrates produced from capture fisheries	Intercept	-7385.5%	1611.2%	-4.58	1.35e-5
	Year	3.9%/year	0.80%/year	4.82	5.31e-6
Nutrients from fishes produced from mariculture (excluding Omega-3 fatty acid)	Intercept	-10530%	2376.0%	-44.31	<2e-16
	Year	53.7%/year	1119%/year	44.71	<2e-16
Nutrients from fishes produced from mariculture (Omega-3 fatty acid)	Intercept	-2.35e5%	693.4%	-33.86	<2e-16
	Year	118.2%/year	3.5%/year	34.14	<2e-16
Nutrients from invertebrates produced from mariculture (excluding Omega-3 fatty acid)	Intercept	-1.1e5%	3871%	-27.25	<2e-16
	Year	53.1%/year	1.9%/year	27.48	<2e-16
Nutrients from invertebrates produced from mariculture (Omega-3 fatty acid)	Intercept	-92689.6%	3590.5%	-24.5	<2e-16
	Year	-46.7%/year	1.9%/year	24.7	<2e-16

Table S2. Projected changes in the maximum potential availability of calcium, iron, omega-3-fatty acid and protein from fisheries catches by 2050 (average of 2041 – 2060) relative to 2010 (average of 1991 – 2010) based on the estimated mean nutrient content for taxonomic groups of fishes are used under SSP5-RCP8.5. Ranges represent projections driven by the outputs from the two Earth system models. Small: <30 cm total length, medium: 30 to 60 cm total length, large: >60 cm total length.

	**Changes in nutrient availabilities by 2050 relative to 2000 (%)**				
**Functional groups**	**Calcium**	**Iron**	**Omega-3-fatty acid**	**Protein**	**Proportion of catches in 2000***
Small pelagic fishes	-25.1 to -22.6	-23.9 to -20.7	-23.1 to -18	-21.5 to -18.5	19.1
Medium pelagic fishes	-27.8 to -12.5	-21.5 to -11.4	-10.8 to -9.4	-17.3 to -10.8	17.7
Large pelagic fishes	-23.2 to -14.8	-28.4 to -17.6	-24.2 to -14.8	-23.7 to -14.2	5.1
Small demersal fishes	-9 to -3.2	-9.1 to -3.4	-8.4 to 0.1	-8.6 to -2.7	17.6
Medium demersal fishes	-0.2 to 5.7	0.2 to 8	11.1 to 27.5	4.3 to 12.2	5.9
Large demersal fishes	-5.8 to -5.5	-5.9 to -4.3	-2.6 to 12	-3.7 to 4.4	2.5
Small benthopelagic fishes	0.4 to 3.6	3 to 5	1.6 to 17.3	3.6 to 21.1	0.2
Medium benthopelagic fishes	2.2 to 19.5	0.1 to 16	6 to 26.1	4.2 to 24.8	3
Large benthopelagic fishes	1.6 to 3.8	3.5 to 5.6	9.2 to 14.9	6.3 to 10.3	10.6
Small reef-associated fishes	2.2 to 14.1	1.5 to 10.3	-3.8 to -3.2	0.9 to 12.1	0.4
Medium reef associated fishes	6 to 112.3	21.7 to 232.2	0 to 39.1	4.8 to 84.7	1.2
Large reef associated fishes	0.4 to 1.4	0.7 to 8.8	-4.9 to -3.6	-1.5 to -0.9	0.2
Small to medium flatfishes	-3.3 to -3.1	-3.9 to -2.3	-4.3 to -2.7	-4.9 to -2.7	1.1
Large flatfishes	-5.1 to -2.9	-3.5 to -0.2	-0.2 to 4.1	-3.9 to -0.7	0.2
Small bathypelagic fishes	-23.1 to -9.9	-23.1 to -9.9	-23.2 to -9.9	-22.5 to -9.7	< 0.1
Medium bathypelagic fishes	36.2 to 93.1	55.8 to 109.4	21.3 to 84.3	41.3 to 101	0.2
Large bathypelagics fishes	-56.9 to -55.1	-57 to -55.9	-57 to -56.5	-57 to -55.9	< 0.1
Small bathydemersal fishes	-12.3 to -12.2	-12.3 to -12.2	-12.3 to -12.2	-12.3 to -12.2	< 0.1
Medium bathydemersal fishes	-9.1 to -2.3	-9.3 to 2.4	-9.7 to 0.8	-9.2 to 4.3	0.2
Large bathydemersal fishes	-4.2 to -0.2	-4.5 to 0	-3.6 to 0.6	-4.6 to 0.1	0.2
Small to medium sharks	-14.2 to -7.5	-13.8 to -7.5	-7 to -6.8	-12.5 to -7.3	< 0.1

Large sharks	-5.4 to 1.8	-7.7 to -0.1	-1.9 to 4.1	-5.7 to 0.9	0.5
Small to medium rays	-3 to -0.7	-4.4 to -1	-2.5 to 1.9	-7.1 to -2.9	0.5
Large rays	-0.3 to -0.1	-4.5 to -2.7	-3.7 to -3.3	-3.3 to -2.4	0.1
Cephalopods	2.5 to 8.4	2.5 to 8.4	2.5 to 8.4	2.5 to 8.4	4
Shrimps	-5 to -0.8	-5 to -0.8	-5 to -0.8	-5 to -0.8	2.5
Lobsters and crabs	-6 to -1.7	-6 to -1.7	-6 to -1.7	-6 to -1.7	2.4
Other demersal Invertebrates	-5.1 to -5	-4.9 to -4.9	-5.1 to -5	-5.3 to -5	3
Krill	-42.4 to -7.5	-42.4 to -7.5	-42.4 to -7.5	-42.4 to -7.5	0.1

Table S3. Projected changes in the maximum potential availability of calcium, iron, omega-3-fatty acid and protein from fisheries catches by 2100 (average of 2081 – 2100) relative to 2010 (average of 1991 – 2010) based on the estimated mean nutrient content for taxonomic groups of fishes are used under SSP5-RCP8.5. Ranges represent projections driven by the outputs from the two Earth system models. Small: <30 cm total length, medium: 30 to 60 cm total length, large: >60 cm total length.

	Changes in nutrient availabilities by 2100 relative to 2000 (%)				
**Functional groups**	**Calcium**	**Iron**	**Omega-3-fatty acid**	**Protein**	**Proportion of catches in 2000***
Small pelagic fishes	-63.7 to -49	-64.9 to -49.2	-56.5 to -42.5	-59.6 to -43.8	19.1
Medium pelagic fishes	-54.9 to -42.5	-46 to -36	-33.3 to -25.3	-41.4 to -33	17.7
Large pelagic fishes	-51.2 to -41	-55.8 to -47.4	-49.4 to -41.6	-49.9 to -40.5	5.1
Small demersal fishes	-14.9 to -12.7	-15.2 to -12	-12.7 to -11.9	-13.2 to -12.1	17.6
Medium demersal fishes	4.4 to 5.4	6.2 to 9.5	29 to 29.4	19.4 to 22.8	5.9
Large demersal fishes	-13.5 to -0.6	-10.3 to 1.7	-14.3 to 47.7	-11.2 to 26	2.5
Small benthopelagic fishes	48.6 to 66.1	58.6 to 70.3	87.3 to 140.2	102.6 to 159	0.2
Medium benthopelagic fishes	-3.7 to 49.7	-6 to 41.9	-1.8 to 64.8	-2.5 to 62.4	3
Large benthopelagic fishes	2.7 to 8.8	8 to 13.1	11.6 to 22.9	3.1 to 15.5	10.6
Small reef-associated fishes	-6.4 to 12.7	-5.6 to 5.7	-14.4 to -8.9	-8.1 to 11.1	0.4
Medium reef associated fishes	13.3 to 109.5	48.3 to 221.3	-1 to 40.9	10 to 85.6	1.2
Large reef associated fishes	4.7 to 18.7	5.4 to 27.2	-2.9 to 8.3	1.6 to 18.4	0.2
Small to medium flatfishes	-16.2 to -16	-17.4 to -15.3	-15.3 to -13.3	-19.7 to -14.3	1.1
Large flatfishes	-15.7 to 1.1	-8.9 to 0.2	-2.1 to 2.8	-10.1 to 0.1	0.2
Small bathypelagic fishes	-95 to -85.8	-94.8 to -85.6	-95.2 to -86	-91.8 to -83.3	0
Medium bathypelagic fishes	369.4 to 721.5	531.8 to 860.1	239.7 to 637.5	414.9 to 782.7	0.2
Large bathypelagics fishes	-55.3 to -20.6	-55.8 to -20.2	-56.2 to -19.9	-55.8 to -20.1	< 0.1
Small bathydemersal fishes	-35.5 to -17.1	-35.5 to -17.1	-35.5 to -17.1	-35.5 to -17.1	< 0.1
Medium bathydemersal fishes	-8 to -3.6	-7.4 to 3.2	-8.8 to 1.2	-6.9 to 5.8	0.2
Large bathydemersal fishes	-1.9 to 6.1	0 to 7	7.8 to 8.4	-1.3 to 7	0.2
Small to medium sharks	-44.4 to -28.7	-43.1 to -28.6	-27.5 to -18	-38.9 to -28.3	< 0.1

Large sharks	-12.4 to 9.5	-14.9 to 5.4	-6.1 to 15.8	-12.1 to 7.9	0.5
Small to medium rays	-17.9 to -5.1	-16.8 to -4.5	-18.8 to -0.2	-23 to -15.7	0.5
Large rays	-3.5 to 4.2	-6 to -3.8	-13.9 to -5.3	-6.3 to 0.8	0.1
Cephalopods	-4.9 to 3.8	-4.9 to 3.8	-4.9 to 3.8	-4.9 to 3.8	4
Shrimps	-11.3 to -0.7	-11.3 to -0.7	-11.3 to -0.7	-11.3 to -0.7	2.5
Lobsters and crabs	-20.1 to -7.2	-20.1 to -7.2	-20.1 to -7.2	-20.1 to -7.2	2.4
Other demersal Invertebrates	-22.5 to -19.3	-22.4 to -18.8	-22.5 to -19.3	-22.8 to -19.7	3
Krill	-60.2 to -50.3	-60.2 to -50.3	-60.2 to -50.3	-60.2 to -50.3	0.1

Table S4. Projected changes in the availability of calcium, iron, omega-3-fatty acid and protein from mariculture production of studied taxa by 2050 (average of 2041 – 2060) relative to 2000 (average of 1991 – 2010) under SSP5-8.5 scenario. Ranges represent projections driven by the outputs from the two Earth system models. Small: <30 cm total length, medium: 30 to 60 cm total length, large: >60 cm total length.

	**Changes in nutrient availabilities by 2050 relative to 2000 (%)**				
**Functional groups**	**Calcium**	**Iron**	**Omega-3-fatty acid**	**Protein**	**Proportion of production in 2000**
Invertebrates	-5.4 to 5.1	-6 to 3.1	-5.4 to 5.1	-4.6 to 7.5	76.5
Large benthopelagic fishes	-19.4 to -9.6	-34.1 to -22.7	-3.7 to 6.2	-27.2 to -17.2	15.7
Large demersal fishes	-54.4 to -53	-55.1 to -53.2	-54.6 to -52.7	-54.5 to -52.4	4.2
Large pelagic fishes	115.1 to 166.7	127.2 to 185.2	112 to 157.7	113.4 to 162.7	1.1
Medium demersal fishes	17.2 to 31.8	24.6 to 43.7	-23.6 to 3.3	37.2 to 42.8	0.8
Medium pelagic fishes	170.3 to 239.5	171 to 239.7	170.4 to 239.6	172.1 to 239.9	0.7
Large Reef associated fishes	148.2 to 164.2	117.3 to 129.7	48.4 to 52.4	139.8 to 152.5	0.5
Small to medium Flatfishes	293.4 to 303.1	292.8 to 303	292.4 to 303.6	294 to 302.5	0.3
Medium bathydemersal fishes	174.4 to 288.7	174.4 to 288.7	174.4 to 288.7	174.4 to 288.7	0.1
Large flatfishes	-12.4 to 6.2	-34.1 to -26.3	-34.8 to -27.6	-33.8 to -25.8	0.1
Medium benthopelagic fishes	330.7 to 700.6	405.3 to 676.6	238.9 to 712.1	347.8 to 697	< 0.1
Medium reef associated fishes	98 to 134.6	77.5 to 105	132.8 to 139.9	118.5 to 168.2	< 0.1

Table S5. Projected changes in the availability of calcium, iron, omega-3-fatty acid and protein from mariculture production of studied taxa by 2090 (average of 2081 – 2100) relative to 2000 (average of 1991 – 2010) under SSP5-8.5 scenario. Ranges represent projections driven by the outputs from the two Earth system models. Small: <30 cm total length, medium: 30 to 60 cm total length, large: >60 cm total length.

	**Changes in nutrient availabilities by 2100 relative to 2000 (%)**				
**Functional groups**	**Calcium**	**Iron**	**Omega-3-fatty acid**	**Protein**	**Proportion of production in 2000**
Invertebrates	-17.7 to -4.5	-17.7 to -4.9	-17.7 to -4.5	-17.5 to -4	76.5
Large benthopelagic fishes	-37.9 to -7	-46.7 to -24.3	-44.7 to 6.4	-44.3 to -12.8	15.7
Large demersal fishes	-59.6 to -58.5	-60.3 to -58.8	-59.4 to -58.2	-58 to -57.9	4.2
Large pelagic fishes	41.7 to 91.7	57.1 to 103.9	33.8 to 81.4	38.2 to 87.6	1.1
Medium demersal fishes	-33.8 to 9.9	-33.5 to 13.6	-25.3 to 0.1	1 to 19	0.8
Medium pelagic fishes	-37.1 to 202.6	-36.8 to 202.9	-37 to 202.7	-36.5 to 203.3	0.7
Large Reef associated fishes	73.8 to 134	64.6 to 110.8	15.2 to 31.2	79.4 to 138	0.5
Small to medium Flatfishes	23.1 to 73.4	22.4 to 72.1	18.2 to 69.5	27.8 to 76.4	0.3
Medium bathydemersal fishes	8.9 to 48.3	8.9 to 48.3	8.9 to 48.3	8.9 to 48.3	0.1
Large flatfishes	-67.1 to 6.5	-73.8 to -14.3	-74 to -15.1	-73.7 to -14	0.1
Medium benthopelagic fishes	220 to 510.7	221.8 to 326.5	219.1 to 737.4	220.2 to 468.5	< 0.1
Medium reef associated fishes	44.3 to 112.1	36.8 to 89.2	49.5 to 121.2	53 to 137.4	< 0.1

Table S6. Estimates of edible portions from three sets of studies: FAO (1989)[1], FAO (2016)[2], the Aquatic Foods Composition Database (2021)[3]

	Ufish		FAO		AFCD		Combined		
**Description**	**Spp. (N samples)**	**Edible portion**	**Spp. (N samples)**	**Edible portion**	**Spp. (N samples)**	**Edible portion**	**Min**	**Average**	**Max**
Small Pelagics (<30 cm)	7 (74)	51%	16 (1)	61%	8 (6)	83%	51%	65%	83%
Medium Pelagics (30 - 90 cm)			26 (1)	57%	19 (11)	70%	57%	64%	70%
Large Pelagics (>=90 cm)	7 (37)	42%	5 (1)	59%	10 (6)	88%	42%	63%	88%
Small Demersals (<30 cm)			1 (1)	36%	4 (2)	92%	36%	64%	92%
Medium Demersals (30 - 90 cm)			8 (1)	52%	18 (3)	64%	52%	58%	64%
Large Demersals (>=90 cm)			7 (1)	50%	19 (7)	53%	50%	51%	53%
Small Bathydemersals (<30 cm)					1 (1)	55%	55%	55%	55%
Medium Bathydemersals (30 - 90 cm)					3 (2)	45%	45%	45%	45%
Large Bathydemersals (>=90 cm)					2 (2)	65%	65%	65%	65%
Small Benthopelagics (<30 cm)	1 (12)	36%			4 (1)	59%	36%	47%	59%
Medium Benthopelagics (30 - 90 cm)			4 (1)	51%	8 (7)	57%	51%	54%	57%
Large Benthopelagics (>=90 cm)			4 (1)	58%	14 (9)	61%	58%	60%	61%
Small Reef assoc fish (<30 cm)					1 (1)	50%	50%	50%	50%
Medium Reef assoc fish (30 - 90 cm)			1 (1)	50%	7 (4)	59%	50%	55%	59%
Large Reef assoc fish (>=90 cm)					5 (4)	63%	63%	63%	63%
Large Sharks (>=90 cm)					3 (2)	49%	49%	49%	49%
Small to Medium Rays (<90 cm)					1 (1)	86%	86%	86%	86%
Small to Medium Flatfishes (<90 cm)	1 (3)	33%	4 (1)	49%	6 (3)	36%	33%	39%	49%
Large Flatfishes (>=90 cm)					3 (1)	92%	92%	92%	92%
Cephalopods	7 (18)	78%			4 (3)	80%	78%	79%	80%
Shrimps	20 (37)	48%			14 (4)	56%	48%	52%	56%
Lobsters, Crabs	19 (35)	28%			15 (6)	32%	28%	30%	32%
Other Demersal Invertebrates	21 (52)	34%			26 (6)	34%	34%	34%	34%
Krill					1 (1)	100%	100%	100%	100%

Supplementary figures.

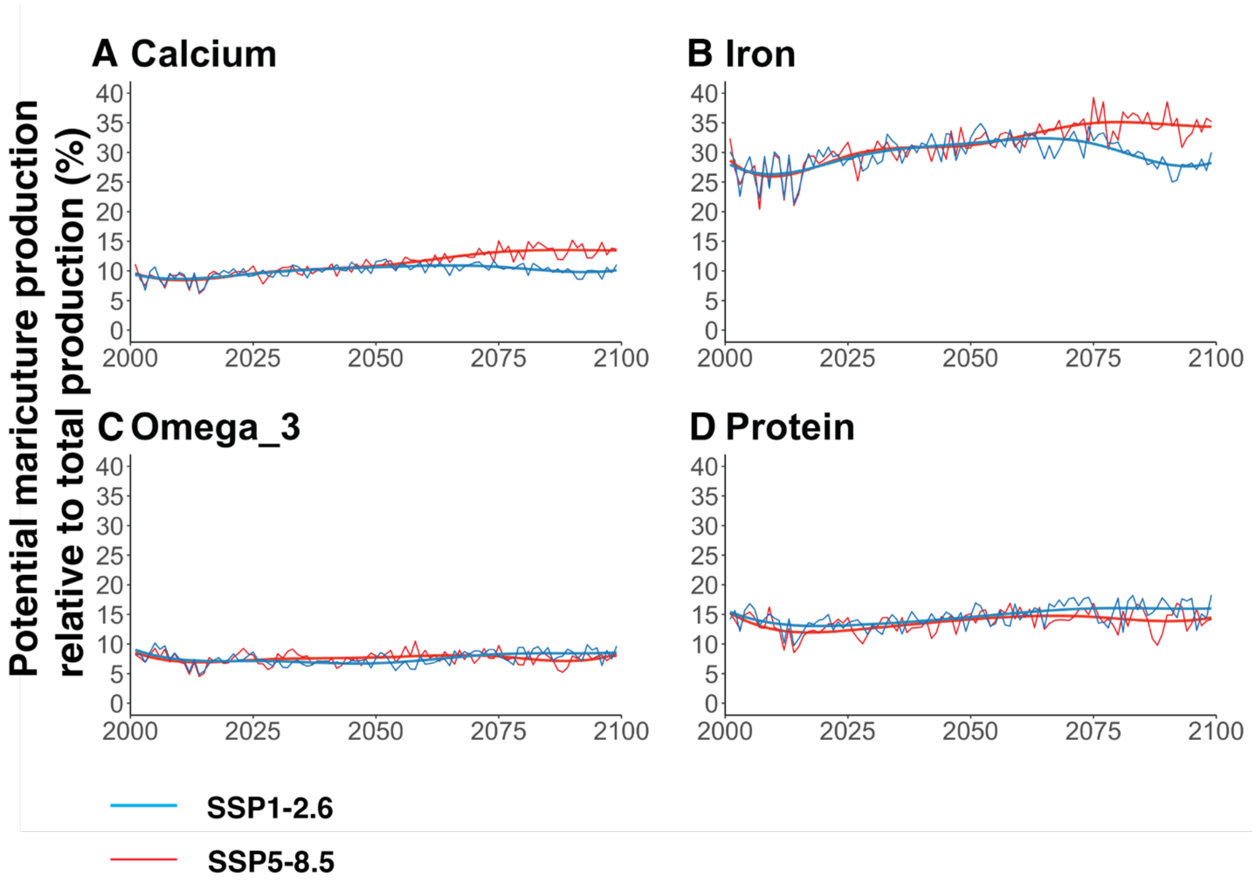

Figure S1. Projected changes in the proportional production of calcium, iron, omega-3 fatty acids and protein from mariculture relative to capture fisheries for the 21st century under climate change scenarios (SSP1-2.6 and SSP5-8.5). The projections were calculated from the mean projections that were driven by different Earth system models.

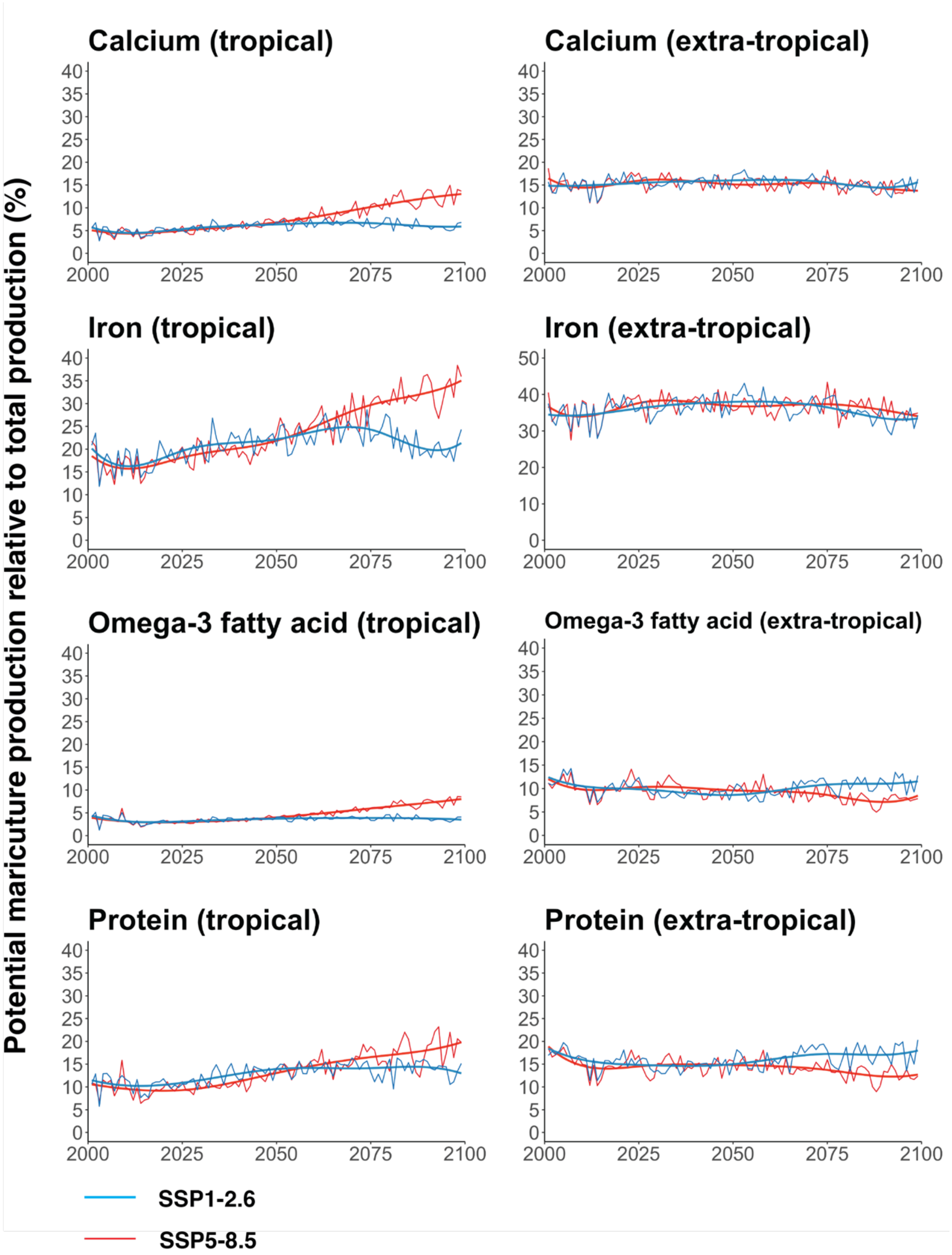

Figure S2. Projected changes in the proportional production of calcium, iron, omega-3 fatty acids and protein from mariculture relative to capture fisheries in tropical (left panels) and extra-tropical (right panels) regions for the 21st century under climate change scenarios (SSP1-2.6 and SSP5-8.5). The projections were calculated from the mean projections that were driven by different Earth system models.
